# Long non-coding RNA CDKN2B-AS1 reduces inflammatory response and promotes cholesterol efflux in atherosclerosis by inhibiting ADAM10 expression

**DOI:** 10.18632/aging.101863

**Published:** 2019-03-29

**Authors:** Haocheng Li, Song Han, Qingfeng Sun, Ye Yao, Shiyong Li, Chao Yuan, Bo Zhang, Bao Jing, Jia Wu, Ye Song, Haiyang Wang

**Affiliations:** 1 Department of Vascular Surgery, The First Affiliated Hospital of Harbin Medical University, Harbin 150001, P. R. China; 2 Department of Cardiac Surgery, The First Affiliated Hospital of Harbin Medical University, Harbin 150001, P. R. China; * Equal contribution

**Keywords:** atherosclerosis, long non-coding RNA CDKN2B-AS1, ADAM10, DNMT1, inflammatory response, cholesterol efflux

## Abstract

Introduction: Long non-coding RNAs (lncRNAs) play key roles in the development of atherosclerosis through the inflammatory pathway. This study aimed to investigate the role of lncRNA cyclin-dependent kinase inhibitor 2B antisense RNA 1 (CDKN2B-AS1) in atherosclerosis via its function in A disintegrin and metalloprotease 10 (ADAM10).

Methods: Initially, the expression of CDKN2B-AS1 and ADAM10 in atherosclerotic plaque tissues and THP-1 macrophage-derived foam cells was determined, after which the cholesterol efflux rate of macrophages was calculated. Interaction between CDKN2B-AS1 and ADAM10 was analyzed, after which, expression of CDKN2B-AS1 and ADAM10 were altered to explore their effects on inflammatory response and cholesterol efflux. The aforementioned findings were further intended to be validated by the atherosclerosis mouse model experiments.

Results: Atherosclerotic plaque tissue and THP-1 macrophage-derived foam cells exhibited downregulated CDKN2B-AS1 and upregulated ADAM10. Upon overexpressing CDKN2B-AS1 or silencing ADAM10, lipid accumulation was reduced and cholesterol efflux was increased. CDKN2B-AS1 located in the nucleus could bind to DNA methyltransferase 1 (DNMT1) to enhance methylation of ADAM10 promoter, leading to suppressed atherosclerotic inflammatory response and promoted cholesterol efflux.

Conclusion: Altogether, lncRNA CDKN2B-AS1 can inhibit the transcription of ADAM10 via DNMT1-mediated ADAM10 DNA methylation, consequently preventing inflammatory response of atherosclerosis and promoting cholesterol efflux.

## Introduction

Atherosclerosis is a chronic vascular disease commonly seen in cardiovascular disease, with its pathogenesis closely linked to the uptake of low-density lipoprotein (LDL) by macrophages and subsequent conversion to foam cells [1]. The risk factors associated with atherosclerosis include smoking, adiposity, hypertension, high blood cholesterol, and diabetes mellitus [2]. The ability of high-density lipoprotein (HDL) to reduce cholesterol from macrophages in the artery wall has been well documented in existing literature with its cardio-protective function highlighted in atherosclerosis [3]. In recent years, the development of atherosclerosis treatment approaches has placed a significant emphasis on inflammation with tremendous progresses achieved [4]. Inflammation suppressors including anti-tumor necrosis factor have been illustrated to be an effective tool capable of inhibiting the pathological processes associated with psoriatic arthritis and carotid atherosclerosis [5]. More importantly, the main obstacle often encountered when treating atherosclerosis comes in the form of the challenge of detecting pre-clinical pathological manifestations [6]. Thus, it is necessary to elucidate the molecular mechanism underlying atherosclerosis in order to identify novel diagnostic and therapeutic modalities capable of more effectively treating atherosclerosis.

Plaque formation represents the pathognomonic hallmark of atherosclerosis caused by the malfunction of endothelial cells that have been shown to be regulated by long non-coding RNAs (lncRNAs) [7]. LncRNAs are composed of more than 200 nucleotides, with studies more recently highlighting their key roles in a variety of cardiovascular diseases and tumors [8]. LncRNA cyclin-dependent kinase inhibitor 2B antisense RNA 1 (CDKN2B-AS1), also known as ANRIL, has been reported to be predominately expressed in tissues associated with coronary heart disease including cardiac, vascular endothelial cells and human monocyte-derived macrophages [9]. Evidence has been presented suggesting that variants of lncRNA CDKN2B-AS1 play critical roles in various diseases including atherosclerosis, diabetes and several types of cancer [10]. In the present study, the interaction between lncRNA CDKN2B-AS1 and a disintegrin and metalloprotease 10 (ADAM10) was determined by an initial methylation-specific PCR (MS-PCR) followed by a confirmatory chromatin immunoprecipitation (CHIP) assay. ADAMs are membrane-bound enzymes that could shed or cleave various cell surface molecules like adhesion molecules, cytokines/chemokines and growth factors. Additionally, reports have implicated ADAMs in a variety of metabolic and inflammatory diseases such as atherosclerosis, diabetes, rheumatoid arthritis [11]. As a member of the integrin and metalloproteinase domain protein family, a close correlation has been identified between ADAM10 and neuro-inflammatory response [12]. A previous study concluded that ADAM10 and ADAM17 are constitutively expressed in lung cells and pivotal shedding enzymes that affect acute lung inflammation [13]. On the other hand, DNA methyltransferase 1 (DNMT1) belongs to the DNA methyltransferase family which can regulate DNA methylation [14]. More importantly, the progress of atherosclerosis is closely associated with pro-inflammatory activation of macrophages i.e. regulated by DNMT [15]. Based on the aforementioned exploration of literature, it was necessary to explore the specific relationship amongst lncRNA CDKN2B-AS1, ADAM10 and DNMT1 and their roles in the pathogenesis of atherosclerosis. Thus, we subsequently tested the hypothesis that the overexpression of lncRNA CDKN2B-AS1 *via* binding to DNMT1 can reduce atherosclerosis and promote cholesterol efflux by downregulating ADAM10.

## RESULTS

### LncRNA CDKN2B-AS1 is downregulated in atherosclerosis

**Figure 1:**
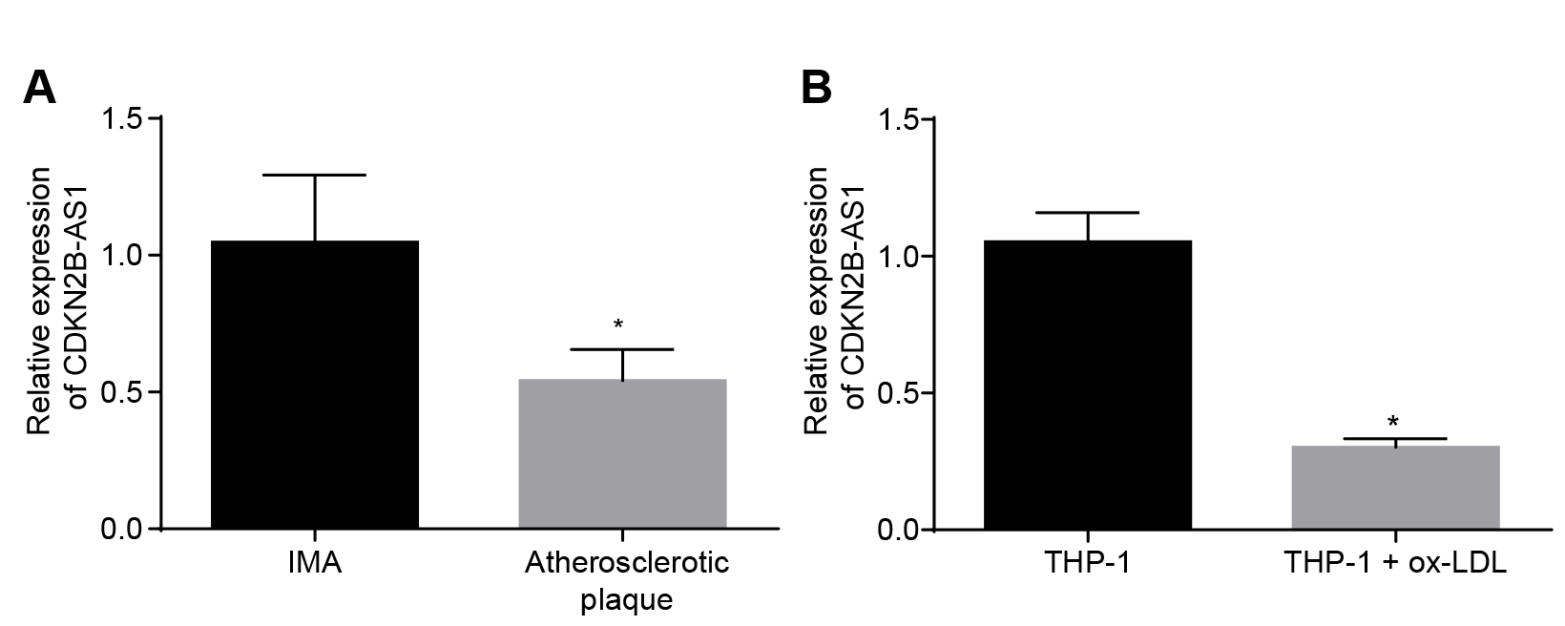
**CDKN2B-AS1 is downregulated in atherosclerosis.** (**A**) RT-qPCR was used to detect the transcriptional level of CDKN2B-AS1 in atherosclerotic plaque and IMA tissues; n = 16; (**B**) RT-qPCR determination of transcription level of CDKN2B-AS1 in ox-LDL-exposed THP-1 macrophage-derived foam cells and THP-1 macrophages; * *p* < 0.05 *vs.* the IMA tissues or THP-1 cells; the measurement data were expressed in the form of mean ± standard deviation and analyzed by unpaired *t*-test, the experiment was repeated 3 times; IMA, internal mammary artery; THP-1, the human monocytic leukemia cell line; RT-qPCR, reverse transcription quantitative polymerase chain reaction; CDKN, cell-dependent kinase inhibitor.

In order to determine the expression of CDKN2B-AS1 in atherosclerosis, both tissue and cellular level expression levels were assessed. RT-qPCR results demonstrated that the relative expression of CDKN2B-AS1 in atherosclerotic plaque tissues was significantly lower than that in non-atherosclerotic internal mammary artery (IMA) tissues (Figure 1A, *p* < 0.05), suggesting that CDKN2B-AS1 is downregulated in atherosclerosis. The expression of CDKN2B-AS1 in human mononuclear cell strain THP-1 macrophage-derived foam cells was also significantly lower than that in THP-1 macrophages (Figure 1B, *p* < 0.05). It was shown that CDKN2B-AS1 was downregulated in THP-1 macrophage-derived foam cells. Based on the above results, it was concluded that CDKN2B-AS1 was poorly expressed in atherosclerosis.

### CDKN2B-AS1 inhibits inflammatory response and promotes cholesterol efflux in atherosclerosis

**Figure 2:**
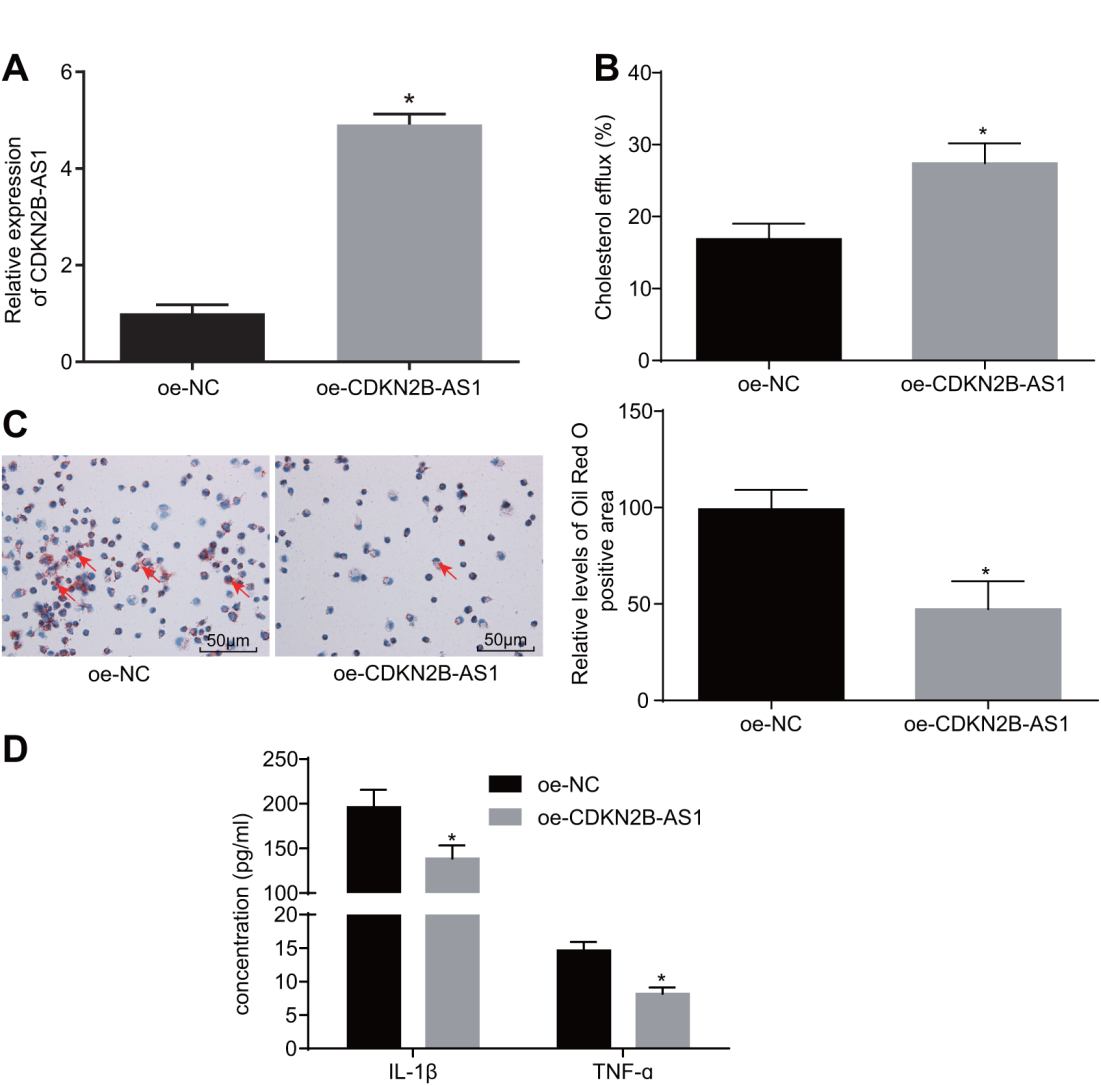
**CDKN2B-AS1 suppresses atherosclerotic inflammatory response and promotes cholesterol efflux.** (**A**) the infection efficiency of lentiviral vector expressing oe-CDKN2B-AS1; (**B**) liquid scintillation counter was used to detect the cholesterol efflux of each group; (**C**) oil red O staining was used to detect the intracellular lipid accumulation of each group (× 200), the red arrows indicate intracellular lipid particles after staining; (**D**) ELISA was used to detect the levels of IL-1β and TNF-ɑ in each group; * *p* < 0.05 *vs.* the oe-NC group; the measurement data were expressed in the form of mean ± standard deviation and analyzed by unpaired *t*-test, the experiment was repeated 3 times; NC, negative control; ELISA, enzyme linked immunosorbent assay; TNF, tumor necrosis factor; IL, interleukin; CDKN, cell-dependent kinase inhibitor.

To further investigate the role of CDKN2B-AS1 in atherosclerosis, a series of experiments were performed. Primarily, the cell lines stably expressing CDKN2B-AS1 were constructed (Figure 2A). The effect of overexpressed CDKN2B-AS1 on cholesterol efflux from THP-1 macrophage-derived foam cells was examined by liquid scintillation counter. Compared with the overexpression-negative control (oe-NC) group, the cholesterol efflux rate of the oe-CDKN2B-AS1 group was increased (Figure 2B, *p* < 0.05), indicating that CDKN2B-AS1 could promote the cholesterol efflux of THP-1 macrophage-derived foam cells. Oil red O staining was also employed in order to determine the effects of CDKN2B-AS1 overexpression on intracellular lipid accumulation in THP-1 macrophage-derived foam cells. This parameter was identified to be more sharply diminished in the oe-CDKN2B-AS1 group than in the oe-NC group (Figure 2C, *p* < 0.05). Consistent with the oil red O staining results, the findings of the high performance liquid chromatography (HPLC) illustrated that total cholesterol (TC), free cholesterol (FC), and cholesterol ester (CE) were significantly decreased in the oe-CDKN2B-AS1 group when compared to the oe-NC group (Table 1, *p* < 0.05). Additionally, the levels of interleukin-1 beta (IL-1β) and tumor necrosis factor-ɑ (TNF-ɑ) in THP-1 macrophage culture medium were also detected by enzyme-linked immunosorbent assay (ELISA). Compared with the oe-NC group, the levels of IL-1β and TNF-ɑ in the oe-CDKN2B-AS1 group were significantly decreased (Figure 2D, *p* < 0.05), which ultimately suggested that CDKN2B-AS1 could inhibit the inflammatory response in atherosclerosis. Taken together, the above results demonstrated that CDKN2B-AS1 can inhibit inflammatory response in atherosclerosis and promote cholesterol efflux.

**Table 1:** HPLC detection of the effect of CDKN2B-AS1 on cholesterol level in THP-1 macrophage-derived foam cells.

Group	TC (μg/mg cell protein)	FC (μg/mg cell protein)	CE (μg/mg cell protein)	CE/TC (%)
oe-NC	465.14 ± 12.34	188.22 ± 10.25	298.24 ± 14.05	64.10
oe-CDKN2B-AS1	289.21 ± 11.02^a^	119.83 ± 9.32 ^a^	162.79 ± 13.05 ^a^	56.38

Note: TC, total cholesterol; FC, free cholesterol; CE, cholesterol ester; ^a^, *p* < 0.05 *vs.* the oe-NC group; the measurement data is expressed in the form of mean ± standard deviation and analyzed by unpaired *t*-test, the experiment was repeated 3 times; THP-1, the human monocytic leukemia cell line; NC, negative control; CDKN, cell-dependent kinase inhibitor.

### ADAM10 is highly expressed in atherosclerosis

**Figure 3:**
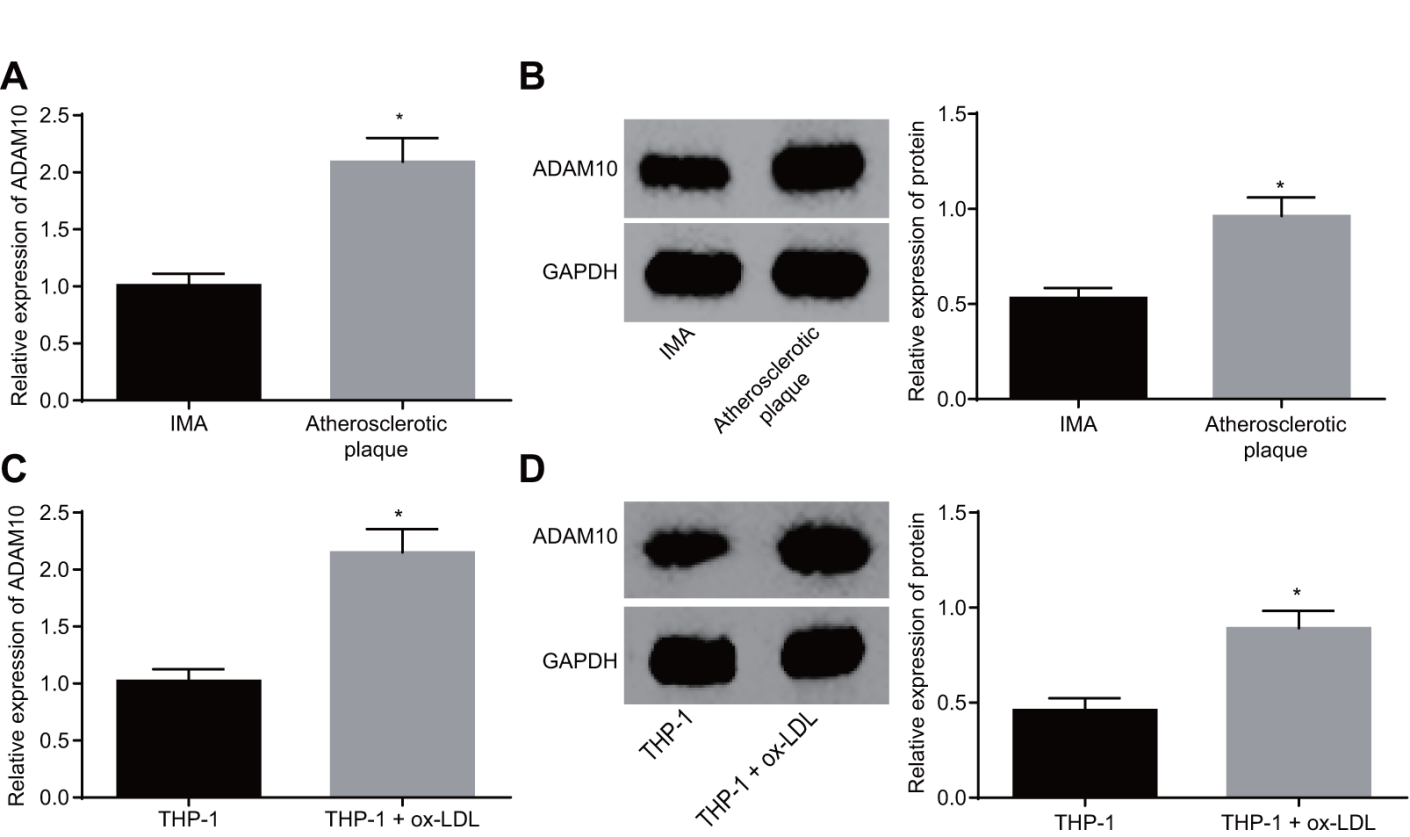
**ADAM10 is upregulated in atherosclerosis.** (**A**) RT-qPCR detects the transcriptional level of ADAM10 in atherosclerotic plaque and IMA tissues; n = 16; (**B**) the protein level of ADAM10 in atherosclerotic plaque and IMA tissues determined by Western blot analysis; n = 16; (**C**) RT-qPCR was used to detect the transcriptional level of ADAM10 in ox-LDL-exposed THP-1 macrophage-derived foam cells and THP-1 macrophages; (**D**) Western blot analysis was used to detect the protein level of ADAM10 protein in atherosclerotic plaque and IMA tissues;* *p* < 0.05 *vs.* the IMA tissues or THP-1 cells; the measurement data were expressed in the form of mean ± standard deviation and analyzed by unpaired *t*-test, the experiment was repeated 3 times; IMA, internal mammary artery; THP-1, the human monocytic leukemia cell line; ADAM10, A disintegrin and metalloprotease 10; RT-qPCR, reverse transcription quantitative polymerase chain reaction; CDKN, cell-dependent kinase inhibitor.

Existing literature has linked relatively high levels of ADAM10 expression with atherosclerosis [11]. In order to identify the role of ADAM10 in atherosclerosis, its expression was investigated at both the tissue and cellular levels. The results of reverse transcription-quantitative polymerase chain reaction (RT-qPCR) and Western blot analysis both revealed significantly higher expression of ADAM10 in atherosclerotic plaque tissues when compared to the non-atherosclerotic IMA tissues (Figure 3A, B, *p* < 0.05). This result indicated that ADAM10 was upregulated in atherosclerosis. The expression of ADAM10 in THP-1 macrophage-derived foam cells was in addition found to be higher than that in the THP-1 macrophages (Figure 3C, D, *p* < 0.05). Based on the above findings, it was concluded that ADAM10 expression was upregulated in THP-1 macrophage-derived foam cells, suggesting that ADAM10 was highly expressed in atherosclerosis.

### Silencing ADAM10 inhibits inflammatory response and promotes cholesterol efflux in atherosclerosis

**Figure 4:**
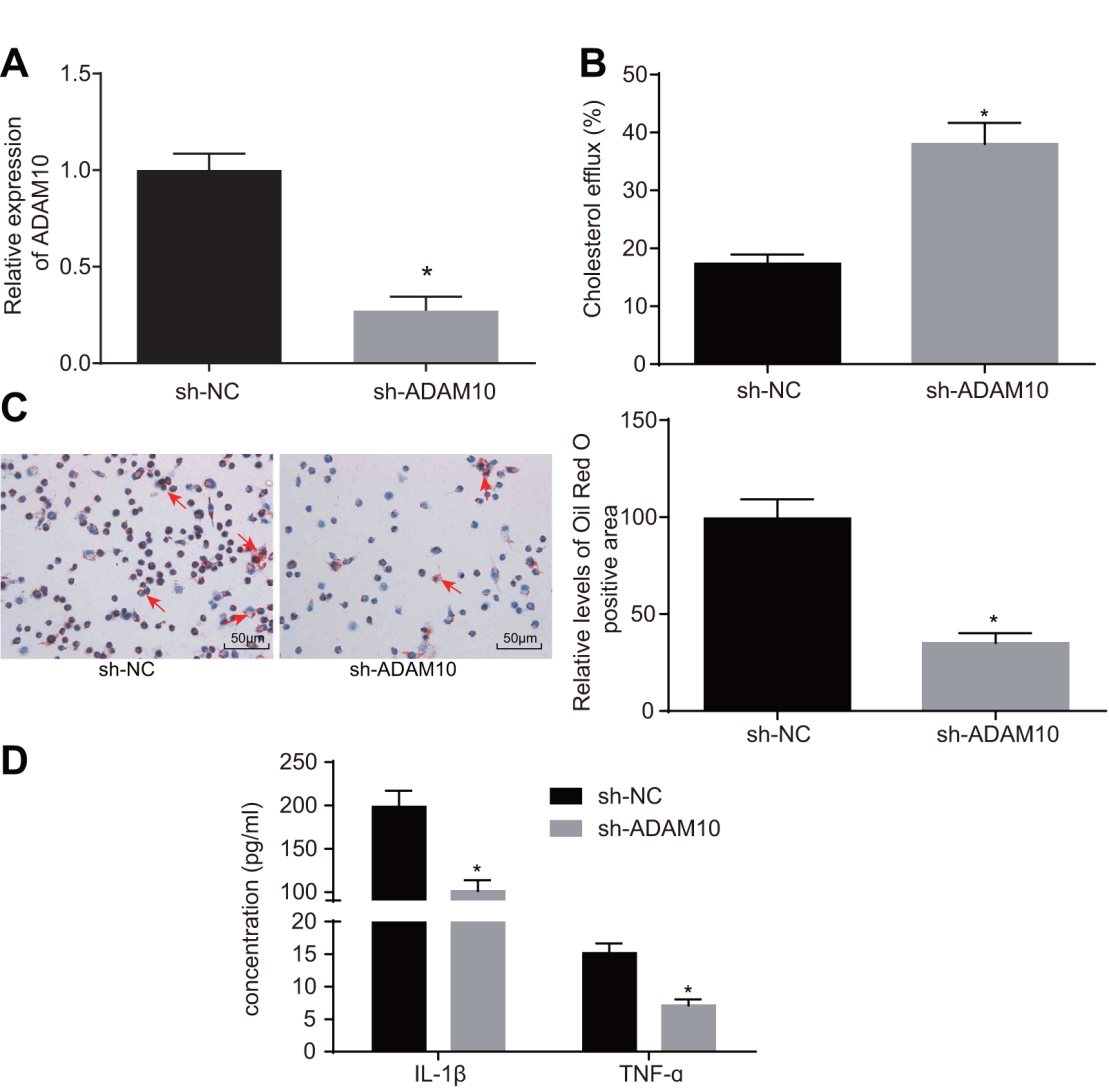
**ADAM10 silencing suppresses inflammatory response and promotes cholesterol efflux in atherosclerosis.** (**A**) the expression of ADAM10 after infection with lentiviral vector expressing sh-ADAM10 determined by RT-qPCR; (**B**) liquid scintillation counter was used to detect the cholesterol efflux of cells infected with lentiviral vector expressing sh-ADAM10; (**C**) oil red O staining was used to detect the lipid accumulation of cells infected with lentiviral vector expressing sh-ADAM10 (× 200), the red arrows indicate intracellular lipid particles after staining; (**D**) ELISA was used to detect IL-1β and TNF-ɑ in cells infected with lentiviral vector expressing sh-ADAM10; * *p* < 0.05 *vs.* the sh-NC group; the measurement data were expressed in the form of mean ± standard deviation and analyzed by unpaired *t*-test, the experiment was repeated 3 times; ELISA, enzyme linked immunosorbent assay; TNF, tumor necrosis factor; IL, interleukin.

To further elucidate the role of ADAM10 in atherosclerosis, the following experiments were performed. Initially, cell lines with stable knockdown of ADAM10 were constructed (Figure 4A). The effects of ADAM10 silencing on cholesterol efflux from the THP-1 macrophage-derived foam cells were examined using a liquid scintillation counter. The rate of cholesterol efflux in the short hairpin RNA (sh)-ADAM10 group exhibited elevated levels when compared to the sh-NC group (Figure 4B, *p* < 0.05); indicating that ADAM10

silencing promoted the cholesterol efflux of THP-1 macrophage-derived foam cells. Oil red O staining was employed in order to detect the effect of ADAM10 silencing on intracellular lipid accumulation in THP-1 macrophage-derived foam cells. Compared with the sh-NC group, intracellular lipid accumulation was reduced in the sh-ADAM10 group (Figure 4C, *p* < 0.05). Consistent with the oil red O staining results, the HPLC results revealed that TC, FC, and CE were significantly decreased in the sh-ADAM10 group compared with that in the sh-NC group (Table 2, *p* < 0.05). ELISA was subsequently employed to determine the levels of IL-1β and TNF-ɑ in the THP-1 macrophage culture medium. Compared with the sh-NC group, the levels of IL-1β and TNF-ɑ in the sh-ADAM10 group were significantly decreased (Figure 4D, *p* < 0.05), suggesting that silencing ADAM10 could inhibit the inflammatory response associated with atherosclerosis. The aforementioned results revealed that silencing ADAM10 can inhibit inflammatory response and promote cholesterol efflux in atherosclerosis.

**Table 2:** HPLC detection of cholesterol levels in THP-1 macrophage-derived foam cells in the sh-NC and sh-ADAM10 groups.

	TC (μg/mg cell protein)	FC (μg/mg cell protein)	CE (μg/mg cell protein)	CE/TC (%)
sh-NC	482.04 ± 13.91	179.15 ± 10.34	306.34 ± 13.28	63.25
sh-ADAM10	285.39 ± 11.24^a^	117.06 ± 9.36^a^	162.62 ± 11.16^a^	57.04

Note: TC, total cholesterol; FC, free cholesterol; CE, cholesterol ester; ^a^, *p* < 0.05 *vs.* the sh-NC group; the measurement data is expressed in the form of mean ± standard deviation and analyzed by unpaired *t*-test, the experiment was repeated 3 times; THP-1, the human monocytic leukemia cell line; NC, negative control; ADAM10, A disintegrin and metalloprotease 10.

### CDKN2B-AS1 promotes methylation of ADAM promoter

**Figure 5:**
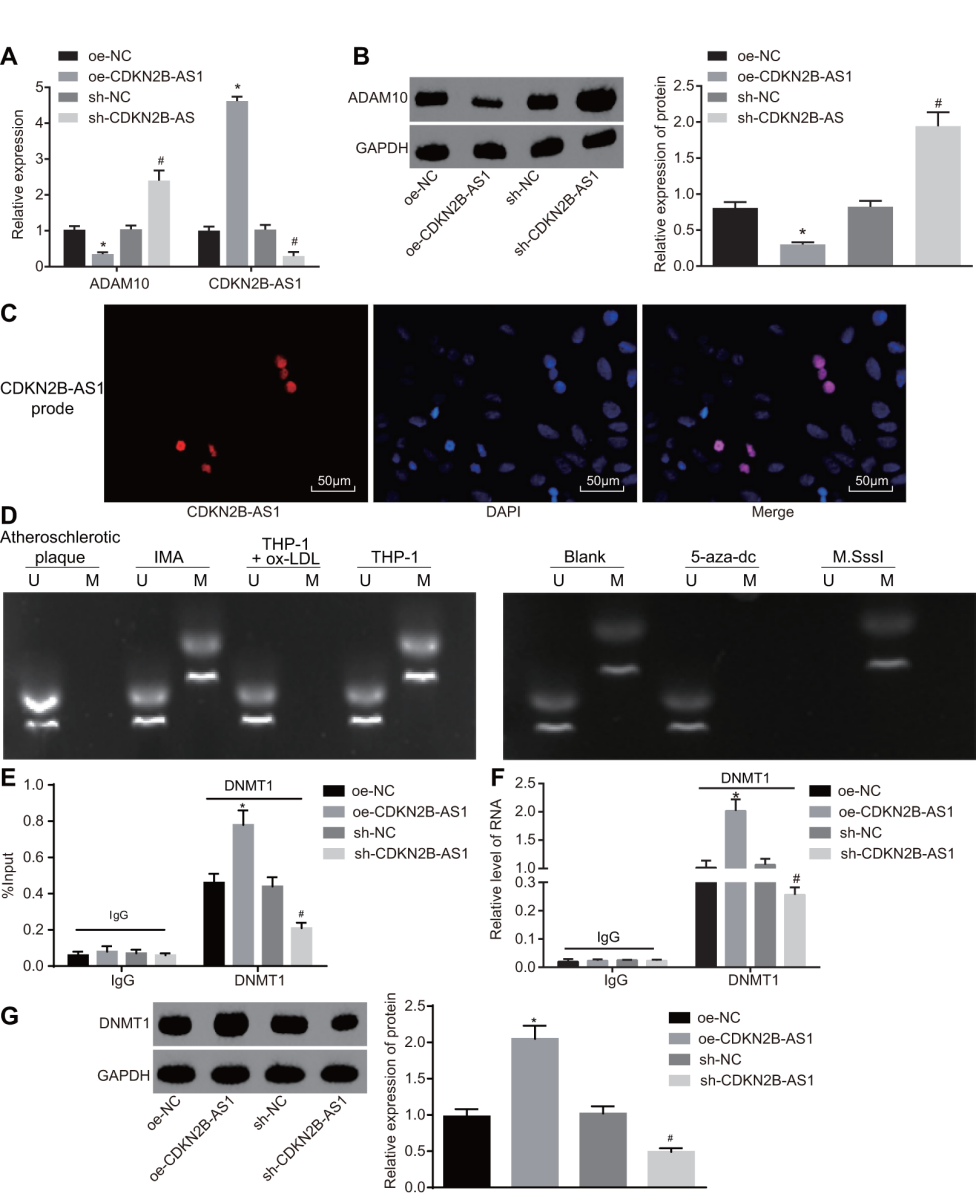
**CDKN2B-AS1 promotes ADAM10 methylation by recruiting DNMT1.** (**A**) the expression of CDKN2B-AS1 and transcription level of ADAM10 in cells infected with lentiviral vector expressing oe-CDKN2B-AS1 or sh-CDKN2B-AS1 determined by RT-qPCR; (**B**) Western blot analysis was used to detect the protein band and level of ADAM10 in cells infected with lentiviral vector expressing oe-CDKN2B-AS1 or sh-CDKN2B-AS1; (**C**) FISH was used to detect CDKN2B-AS1 localization in cells (× 200); (**D**) MS-PCR was used to detect the electrophoresis band of ADAM10 methylation level in atherosclerotic plaque, IMA tissues and ox-LDL-exposed THP-1 macrophages or ox-LDL-exposed THP-1 macrophages treated with 5-aza-dc or M.SssI; (**E**) CHIP assay to detect the output percentage of ADAM10 in cells infected with lentiviral vector expressing oe-CDKN2B-AS1 or sh-CDKN2B-AS1; (**F**) RIP assay to detect the output percentage of CDKN2B-AS1 in cells infected with lentiviral vector expressing oe-CDKN2B-AS1 or sh-CDKN2B-AS1; (**G**) RNA pull down to detect the DNMT1 protein pulled down by lncRNA CDKN2B-AS1 in cells infected with lentiviral vector expressing oe-CDKN2B-AS1 or sh-CDKN2B-AS1; In panel D, U represents the un-methylated lane, and M is representative of the methylation lane; * *p* < 0.05 *vs.* the oe-NC group; # *p* < 0.05 *vs.* the sh-NC group; the measurement data were expressed in the form of mean ± standard deviation and analyzed by one-way ANOVA, the experiment was repeated 3 times; THP-1, the human monocytic leukemia cell line; RT-qPCR, reverse transcription quantitative polymerase chain reaction; CDKN, cell-dependent kinase inhibitor; ANOVA, analysis of variance; ELISA, enzyme linked immunosorbent assay; RIP, RNA-binding protein immunoprecipitation; FISH, fluorescence in situ hybridization; CHIP, chromatin immunoprecipitation; MS-PCR, methylation-specific PCR.

The mRNA and protein expression of CDKN2B-AS1 and ADAM10 in each group was detected by RT-qPCR (Figure 5A) and Western blot analysis (Figure 5B) respectively. The results of which indicated that compared with the oe-NC group, the transcription as well as the protein levels of CDKN2B-AS1 were significantly higher while that of ADAM10 in the oe-CDKN2B-AS1 group was significantly lower (*p* < 0.05); compared with the sh-NC group, the transcription level and protein expression of CDKN2B-AS1 were significantly reduced while those of ADAM10 in the sh-CDKN2B-AS1 group were significantly increased (*p* < 0.05). The obtained results revealed that CDKN2B-AS1 negatively regulated the expression of ADAM10. Considering that CDKN2B-AS1 was downregulated and ADAM10 was highly expressed in patients with atherosclerosis, the following experiments were conducted to investigate the mechanism by which CDKN2B-AS1 could inhibit ADAM10 expression. The cellular localization of CDKN2B-AS1 as detected by fluorescence in situ hybridization (FISH) (Figure 5C) demonstrated that CDKN2B-AS1 was located in the nucleus. MS-PCR was applied in order to detect the methylation status of ADAM10 promoter in tissues and cells (Figure 5D). The results indicated that partial methylation of the ADAM10 promoter at specific sites was found in non-atherosclerotic IMA tissues and THP-1 macrophages. Compared with the blank group, the complete methylation of ADAM10 at specific sites was detected in the M.SssI (methyltransferase) group while no evidence of methylation was observed in the 5-aza-dc (DNA methyltransferase inhibitor) group. The CHIP assay was applied in order to detect the enrichment of DNMT1 (methyltransferase) in the ADAM10 promoter region of each group (Figure 5E). The results suggested that compared with the oe-NC group, the enrichment of DNMT1 in the ADAM10 promoter region was significantly increased in the oe-CDKN2B-AS1 group. The enrichment of DNMT1 in the ADAM10 promoter region of the sh-CDKN2B-AS1 group was significantly decreased in comparison to the sh-NC group, indicating that CDKN2B-AS1 can promote the enrichment of DNMT1 in the ADAM10 promoter region and *vice versa*. The RNA-binding protein immunoprecipitation (RIP) assay further verified the binding of DNMT1 (methyltransferase) to CDKN2B-AS1 in each group (Figure 5F). The results illustrated that the level of DNMT1 binding to CDKN2B-AS1 was significantly higher in the oe-CDKN2B-AS1 group than in the oe-NC group. Additionally, the level of DNMT1 binding to CDKN2B-AS1 was significantly decreased in the sh-CDKN2B-AS1 group when compared to the sh-NC group, confirming previous result. The RNA-pull down assay was then performed to detect the level of DNMT1 (methyltransferase) pulled down by CDKN2B-AS1 in each group (Figure 5G). The results revealed that compared with the oe-NC group, the level of DNMT1 (methyltransferase) pulled down by CDKN2B-AS1 in the oe-CDKN2B-AS1 group was significantly increased. Compared with the sh-NC group, the level of DNMT1 pulled down by CDKN2B-AS1 was diminished in the sh-CDKN2B-AS1 group, suggesting that CDKN2B-AS1 can promote CDKN2B-AS1 binding to DNMT1, and silencing CDKN2B-AS1 can obtain the opposite results, which were consistent with the RIP experiment. Taken together, CDKN2B-AS1 can interact with DNMT1 and recruit DNMT1 binding to ADAM10 promoter region, in turn promoting methylation and inhibition of ADAM10 expression.

### Overexpression of CDKN2B-AS1 methylates ADAM10, inhibits atherosclerotic inflammatory response, and promotes cholesterol efflux

**Figure 6:**
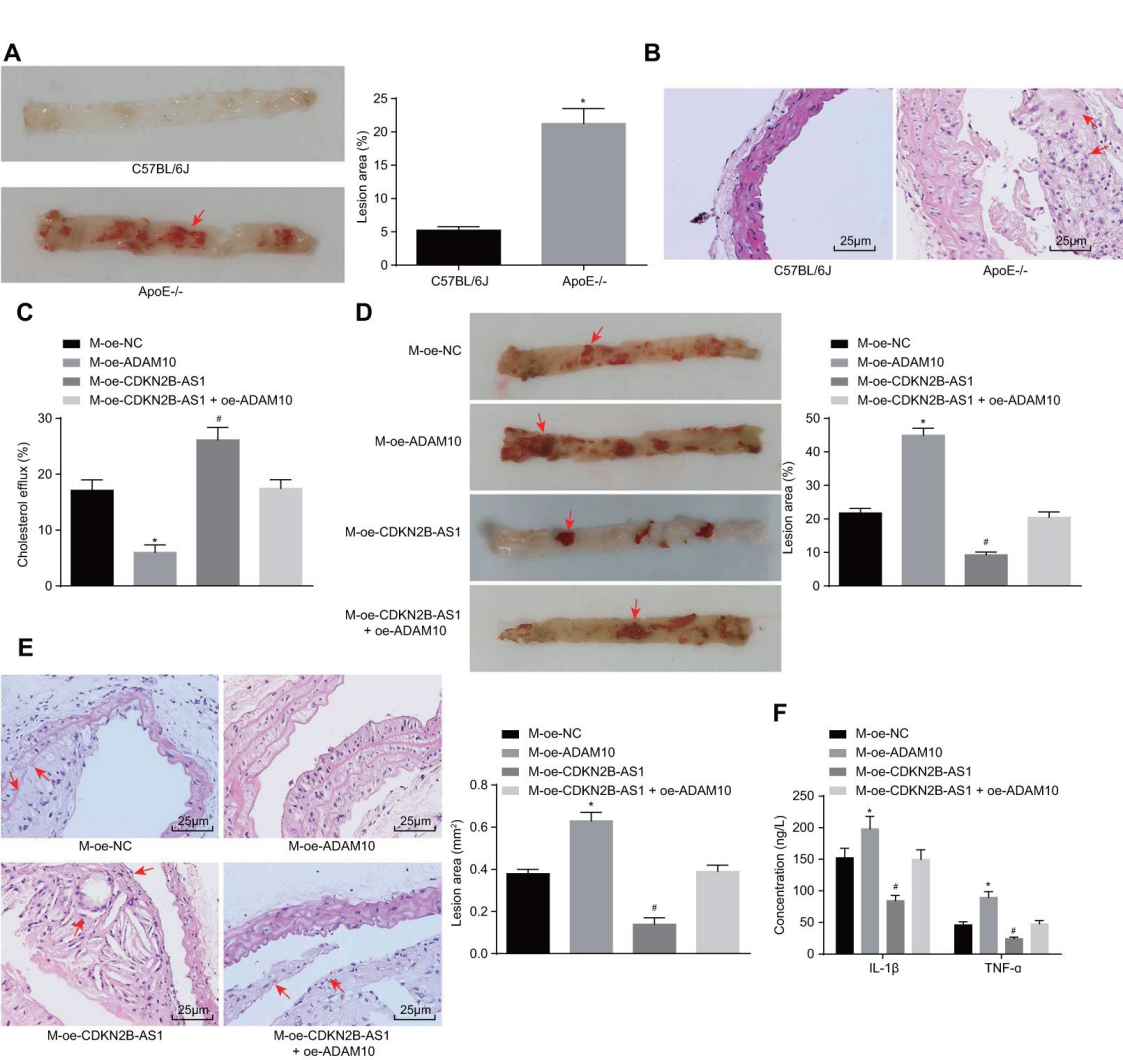
**Overexpression of CDKN2B-AS1 methylates ADAM10, inhibits inflammatory response, and promotes cholesterol efflux in atherosclerosis.** (**A**) Oil red O staining of aortic was used to detect aortic plaque formation in C57BL/6J and ApoE-^/-^ mice (× 10), the red arrows indicate the formed atheromatous plaque; (**B**) HE staining was used to detect arterial plaque formation in C57BL/6J and ApoE-^/-^ mice (× 400), the red arrows indicate atheromatous plaque accompanied by foam cells after HE staining; (**C**) Liquid scintillation counter was applied to measure the effect of various intervention factors on serum cholesterol efflux in ApoE^-/-^ mice; (**D**) Oil red O staining of aortic detection of intervention factors of aortic plaque formation on ApoE^-/-^ mice (× 10), the arrows indicate the formed atheromatous plaque; (**E**) HE staining of aortic roots for detection of aortic plaque formation in ApoE^-/-^ mice (× 400). (**F**) ELISA was used to detect the serum levels of IL-1β and TNF-ɑ in ApoE^-/-^ mice; * *p* < 0.05 the M-oe-ADAM10 group versus the M-oe-NC group; # *p* < 0.05 the M-oe-CDKN2B-AS1 group versus the M-oe-NC group; the measurement data were expressed in the form of mean ± standard deviation and analyzed by unpaired *t*-test, n = 12; the experiment was repeated 3 times; HE, hematoxylin-eosin; ELISA, enzyme linked immunosorbent assay; TNF, tumor necrosis factor; IL, interleukin; CDKN, cell-dependent kinase inhibitor; ADAM10, A disintegrin and metalloprotease 10.

In order to further investigate the effects associated with CDKN2B-AS1 and ADAM10 on the progression of atherosclerosis *in vivo*, the ApoE knockout mice recruited for the purposes of the study were placed on a high-fat diet for 10 weeks in order to establish the animal models of atherosclerosis. The modeling success rate was determined to be 96%. The mice that were successfully established were dark and dull, pale and fatigued with loss of appetite, in addition to exhibiting slow movements. The blood lipid levels of the control group and the model group demonstrated that the levels of TC, triglyceride (TG), HDL and LDL in the ApoE^-/-^ mice were significantly higher than those in the C57BL/6J mice (*p* < 0.05) (Table 3). Oil red O staining of the aorta showcased distinct atherosclerotic plaque in the aortic arch, abdominal aorta, thoracic aorta and common iliac artery branch of ApoE^-/-^ mice after induction by high-fat diet. The lipids in the atherosclerotic plaque were also stained with oil red O, while the normal C57BL/6J mice had no obvious atherosclerotic plaque formation (Figure 6A). The results of hematoxylin-eosin (HE) staining in the aortic root were similar to those of the oil red staining of aorta. In the ApoE^-/-^ mice induced by the high-fat diet, the blood vessel wall of the aortic root was thickened, and the foam cells were accumulated, partially ruptured, and distinct atherosclerotic lesions were observed. There were no atherosclerotic changes including foam cell formation in the aortic root of C57BL/6J mice, with the cell morphology found to be normal and arranged closely (Figure 6B). Taken together, the high-fat diet-induced ApoE^-/-^ mice showed typical pathological features of atherosclerosis which were then employed for subsequent experiments. The cholesterol efflux rate of the M-oe-CDKN2B-AS1 group was significantly higher than that of the M-oe-NC group by detecting the body cholesterol efflux rate among each group (*p* < 0.05). The cholesterol efflux rate of the M-oe-ADAM10 group was significantly decreased (*p* < 0.05) while no significant difference was detected regarding the cholesterol efflux rate of the M-oe-CDKN2B-AS1 + oe-ADAM10 group (*p* < 0.05) (Figure 6C), indicating that overexpression of CDKN2B-AS1 promoted cholesterol efflux and overexpression of ADAM10 inhibited cholesterol efflux. Thus, it was concluded that overexpression of CDKN2B-AS1 could reverse the effect of ADAM10 on cholesterol efflux *in vivo*. Additionally, oil red O staining of aorta in Figure 6D showed attenuated atherosclerotic lesions in the M-oe-CDKN2B-AS1 group when compared with the M-oe-NC group (*p* < 0.05), while more severely aggravated lesions were detected in the M-oe-ADAM10 group (*p* < 0.05). Meanwhile the M-oe-CDKN2B-AS1 + oe-

**Table 3:** Blood lipid levels of mice in each group (mmol/L).

Group	TC	TG	HDL	LDL
C57BL/6J	3.17 ± 0.17	1.05 ± 0.21	2.14 ± 0.13	0.82 ± 0.11
ApoE^-/-^	27.3 ± 0.87*	8.42 ± 0.28*	11.23 ± 0.76*	14.39 ± 0.56*

Note: TC, total cholesterol; TG, triglycerides; HDL, high density lipoprotein; LDL, low density lipoprotein; *, *p* < 0.05 *vs.* the C57BL/6J group; n = 12 (the C57BL/6J mice); n = 48 (ApoE^-/-^); Data between two groups was compared using *t*-test.

ADAM10 group exhibited no significant changes regarding atherosclerotic lesions, indicating that overexpression of CDKN2B-AS1 inhibited atherosclerotic lesions, overexpression of ADAM10 promoted atherosclerosis in sclerotic lesions, while the overexpression of CDKN2B-AS1 reversed the effects of ADAM10 on atherosclerotic lesions. The HPLC results (Table 4) indicated that intracellular TC, FC, and CE were significantly decreased in the M-oe-CDKN2B-AS1 group than those in the M-oe-NC group (*p* < 0.05), while significant increases were observed in the M-oe-ADAM10 group (*p* < 0.05). No significant changes in relation to the aforementioned parameters were observed in the M-oe-CDKN2B-AS1 + oe-ADAM10 group. These findings indicated that overexpression of CDKN2B-AS1 inhibited intracellular lipid accumulation, overexpression of ADAM10 promoted intracellular lipid accumulation, while the overexpression of CDKN2B-AS1 reversed the effect of ADAM10 on intracellular lipid accumulation. Furthermore, HE staining results in Figure 6E revealed that atherosclerotic plaque was reduced in the M-oe-CDKN2B-AS1 group compared with the M-oe-NC group (*p* < 0.05) while it was elevated in the M-oe-ADAM10 group (*p* < 0.05). There were no significant changes in the M-oe-CDKN2B-AS1 + oe-ADAM10 group, indicating that overexpression of CDKN2B-AS1 inhibited atherosclerosis, overexpression of ADAM10 promoted atherosclerosis, and overexpression of CDKN2B-AS1 can reverse the effect of ADAM10 on atherosclerosis. The serum levels of IL-1β and TNF-ɑ in ApoE^-/-^ mice were measured by ELISA (Figure 6F). AS compared with the M-oe-NC group, the serum levels of IL-1β and TNF-ɑ in the M-oe-CDKN2B-AS1 group were significantly lower (*p* < 0.05) while the serum levels of IL-1β and TNF-ɑ were elevated in the M-oe-ADAM10 group (*p* < 0.05). No significant changes in relation to the serum levels of IL-1β and TNF-ɑ in the M-oe-CDKN2B-AS1 + oe-ADAM10 group were detected, highlighting the inhibitory role of CDKN2B-AS1 in the stimulating effect of ADAM10 on the inflammatory response associated with atherosclerosis.

**Table 4:** HPLC detection of cholesterol levels in peritoneal macrophages of mice in each group (g/mg cell protein).

	TC	FC	CE	CE/TC (%)
M-oe-NC	489.14 ± 18.26	187.72 ± 29.06	302.91 ± 28.15	62.03
M-oe-CDKN2B-AS1	408.24 ± 32.27 ^a^	106.34 ± 26.65 ^a^	212.82 ± 33.61 ^a^	52.50
M-oe-ADAM10	607.21 ± 34.19 ^a^	263.36 ± 21.28^a^	384.84 ± 35.08 ^a^	63.51
M-oe-CDKN2B-AS1 +oe-ADAM10	503.25 ± 21.22	189.34 ± 31.38	314.64 ± 27.16	62.70

Note: TC, total cholesterol; FC, free cholesterol; CE, cholesterol ester; ^a^, *p* < 0.05 *vs.* the M-oe-NC group; all experiments were repeated 3 times; one-way ANOVA was used for data analysis between groups; CDKN, cell-dependent kinase inhibitor; ADAM10, A disintegrin and metalloprotease 10; HPLC, high performance liquid chromatography; ANOVA, analysis of variance.

## DISCUSSION

Atherosclerosis is a chronic inflammatory disease that damages vessel walls [2]. Significant progress has been made regarding the development of treatment aimed at managing patients with the disease, however the greater majority of said therapeutic options with a pharmacological approach are often accompanied by chronic side effects [16]. Thus, it is of great importance to find more effective and long-term treatment for patients with atherosclerosis. A previous study highlighted the potential of lncRNAs could be novel players in atherosclerosis [17] Existing literature has suggested that ADAM10, a mediator of endothelial cell function regulated by vascular endothelial growth factor, is closely related to the development of atherosclerosis [18]. Hence, the current study set out to investigate the effects associated with the lncRNA CDKN2B-AS1 as well as ADAM10 on atherosclerosis. Key observations indicated that overexpressed CDKN2B-AS1 could potentially promote cholesterol efflux in atherosclerosis by downregulating ADAM10 via DNMT1-mediated ADAM10 DNA methylation.

The initial findings of the study revealed that CDKN2B-AS1 was downregulated in atherosclerotic plaque tissues and ox-LDL-exposed THP-1 macrophages. Certain lncRNAs have been shown to play crucial roles in the development and progression of cardiovascular disorders, such as myocardial infarction and atherosclerosis [19,20]. For instance, variants in lncRNA CDKN2B-AS1 have been implicated in the development of coronary artery disease [21–24]. Moreover, studies have highlighted variants in CDKN2B-AS1 (ANRIL) and identified a relationship with atherosclerosis [25,26]. Of crucial importance, the current study obtained evidence verifying the existence of low expression of CDKN2B-AS1 in clinical atherosclerotic plaque tissues, while elucidating the potential anti-atherogenic role of CDKN2B-AS1. In line with our study, it has been reported that the susceptibility to coronary atherosclerosis and the prognosis of disease progression involve extracellular matrix metabolism and fibrogenesis whereby CDKN2B-AS1 assumes an essential role [27]. Additionally, the results of the current study also indicated that ADAM10 was highly expressed in atherosclerotic plaque tissues and ox-LDL-exposed THP-1 macrophages. Many ADAMs have been linked with both the early and advanced stages of atherosclerosis, an example of which ADAM10 elevation was found in unstable atherosclerotic plaque tissues [28,29]. Consistently, a previous study concluded that the abnormal expression of ADAM10 plays a vital role in the progression of atherosclerosis [30] with reports implicating it in the macrophage inflammatory process [31]. The data collected during our study further identified a relationship whereby the overexpression of lncRNA CDKN2B-AS1 induced ADAM10 gene methylation to inhibit its expression, highlighting an underlying regulatory mechanism of CDKN2B-AS1 in atherosclerosis.

The present study also identified that lncRNA CDKN2B-AS1 recruited DNMT1 to ADAM10 promoter region, initiated methylation and inhibited ADAM10 expression, thereby diminishing atherosclerosis and promoting cholesterol efflux. A previous study concluded that rs653765 is a functional variant in the promoter region of ADAM10, while indicating the existence of a close association with the progression of sepsis [32]. Moreover, ADAM10 in leukocyte is associated with inflammation, thus exerting its effects on diminishing recruitment of inflammatory cells [33]. Evidence has been presented suggesting that enforced expression of DNMT1 may lead to the abnormal DNA methylation of genes in gliomas [34]. Similarly, it has been reported that lncRNA Kcnq1ot1 could bind to DNMT1 and then recruit DNMT1, leading to enhanced methylation in CpG island methylation which is specific to paternal [35]. As illustrated by a previous study, atherosclerosis can be suppressed by silkworm protein 30Kc6 with TC, TG and LDL all exhibiting downregulated levels [36]. The overexpression of CDKN2B-AS1 during the current study was observed to result in a decreased in the expression of ADAM10 while the restoration of ADAM10 reversed the CDKN2B-AS1-induced inhibition effects on the levels of TC, TG and LDL, indicating that CDKN2B-AS1 could inhibit atherosclerosis by inhibiting the expression of ADAM10.

Furthermore, a previous report revealed that both IL-1β and TNF-ɑ to be proinflammatory mediators that play crucial roles in the development of atherosclerotic lesions [37]. Concurrently, the levels of IL-1β and TNF-ɑ in the oe-CDKN2B-AS1 group exhibited significant decreases, suggesting that CDKN2B-AS1 could inhibit the atherosclerotic inflammatory response. At the same time, the development of atherosclerosis is closely associated with immune system in which both miRNAs and lncRNAs play important roles [38]. Reports have suggested that lncRNAs are capable of regulating the function of vessel wall, lipid metabolism, activation of macrophages as well as the inflammatory response [39]. On the other hand, genome-wide association studies have demonstrated that CDKN2B-AS1 contains multiple markers of coronary artery disease [40] with reports indicating the destruction of the CDKN2B gene in tumors, highlighting its vital role in cell cycle regulation [41]. ADAM10 downregulation by fish oil has been shown to slow down the development of atherosclerosis in male mice [42]. Existing literature has suggested that ligand-activated Liver X Receptor plays a vital role in increasing cholesterol efflux from cells, while indicating that the expression of ADAM10 can be decreased by inactivation of ligand-activated Liver X Receptor leading to a reduction in the levels of cellular cholesterol [43]. Consistent with the aforementioned literature, our study determined that the overexpression of CDKN2B-AS1 can decrease the expression of ADAM10, inhibit the progression of atherosclerosis and promote cholesterol efflux via binding to DNMT1.

**Figure 7:**
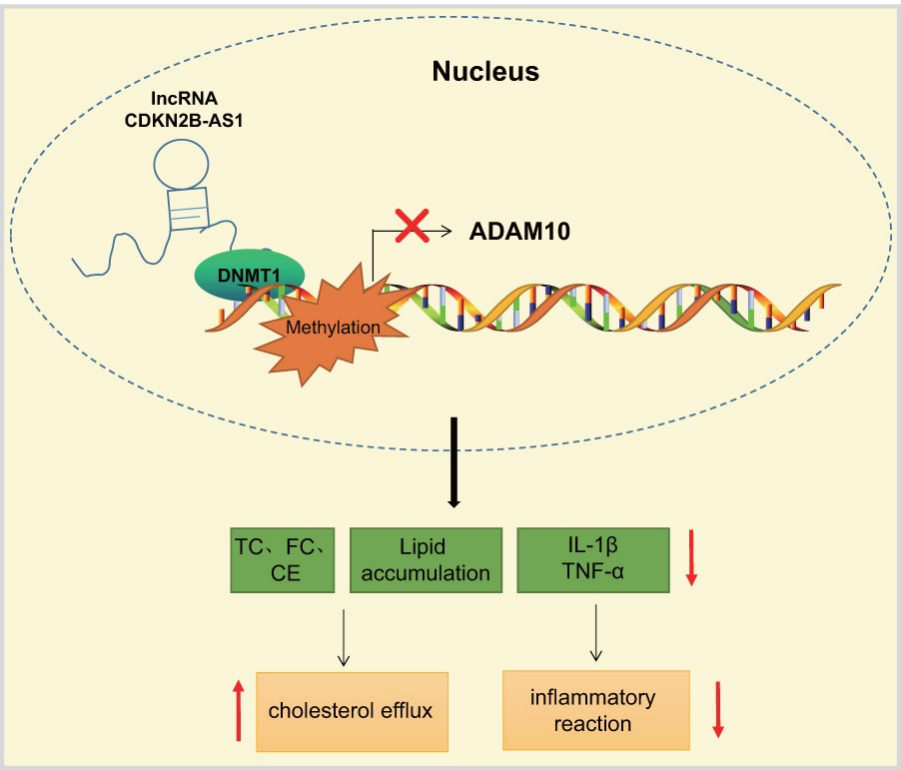
LncRNA CDKN2B-AS1 inhibits the transcription of ADAM10 *via* DNMT1-mediated ADAM10 DNA methylation, consequently preventing inflammatory response of atherosclerosis and promoting cholesterol efflux.

The current study presents evidence confirming that the overexpression of lncRNA CDKN2B-AS1 could inhibit the progression of atherosclerosis by downregulation of ADAM10. CDKN2B-AS1 recruited DNMT1 to ADAM10 promoter region to induce DNA methylation, thereby inhibiting ADAM10 expression (Figure 7). These findings highlight the potential of the lncRNA CDKN2B-AS1, ADAM10 and DNMT1 as potential therapeutic targets for atherosclerosis treatment. However, there were certain limitations faced during the study owing to the small sample size. Therefore, further studies with a lager sample size are required, in order to perform DNMT1-dependent experiments in follow-up studies and exclude other factors mediating CDKN2B-AS1 to ADAM10.

## MATERIALS AND METHODS

### Ethics statement

The study was conducted in accordance with the Institutional Review Board of the First Affiliated Hospital of Harbin Medical University as well as the Helsinki Declaration. All participants signed written informed consent documentation. The animal experiments were conducted in strict accordance with the recommendations in the Guide for the Care and Use of Laboratory Animals. The experimental protocol was approved by the Laboratory Animal Ethics Committee.

### Study subjects

Sixteen patients with coronary heart disease (including 10 males and 6 females with an average age of 57.75 ± 11.08 years) who had previously undergone coronary endarterectomy and bypass grafting in the First Affiliated Hospital of Harbin Medical University between June 2016 and December 2017 were recruited for the purposes of the study. The endometrial tissue of the left anterior descending coronary artery of the atherosclerotic coronary artery was subsequently collected. From the same patient, non-atherosclerotic IMA tissues were obtained as a NC during bypass surgery. A histopathological analysis of atherosclerosis and IMA were conducted in order to confirm the presence or absence of atherosclerosis, respectively. The inclusion criteria were as follows: patients with coronary heart disease were diagnosed by coronary angiography and experienced chest pain symptoms. The exclusion criteria were as follows: patients with other complications such as heart failure, renal failure, liver failure, blood system diseases, malignant tumors, diabetes and infectious diseases.

### Cell culture and transfection

The human mononuclear cell strain (THP-1) was purchased from the Basic Medical Cell Center of the Institute of Basic Medical Sciences of the Chinese Academy of Medical Sciences (Beijing, China) (http://www.crcpumc.com/pr.jsp?keyword=THP1&_pp=0_312). THP-1 monocytes were cultured using Roswell Park Memorial Institute (RPMI) 1640 medium (Gibco, Carlsbad, CA, USA) containing 10% fetal bovine serum (FBS). Next, the THP-1 cells were incubated with 20 μL of 100 nmol/L phorbol myristic acetate (PMA; Sigma-Aldrich, SF, CA, USA) in a six-well plate for 72 h at 37°C and 5% CO_2_ in order to facilitate the growth of the THP-1 cells into a single layer with the macrophages derived from monocytes. The cells were transfected after the macrophages had been induced to foam cells by culturing in serum-free RPMI 1640 medium containing 0.3% bovine serum albumin (BSA) (HyClone Company, Logan, UT, USA) and 50 μg/mL oxidized LDL (ox-LDL) for 48 h.

The vector used to construct the lentiviral vector was pGMLV-PE2 (component sequence: CMV-MCS-eGFP-PGK-puromycin, overexpression vector, oe-), and pGMLV-SC6 (component sequence: hU6-MCS-CMV-RFP-PGK-puromycin, gene silencing vector, sh-), with all these vectors purchased from GenePharma Ltd. (Shanghai, China). The shRNA or gene sequence was cloned into lentivirus vector and then purified. Lentiviral packaging was performed using the 293T cells and cultured in the RPMI-1640 complete medium containing 10% FBS and sub-cultured every other day. The virus was collected and divided into THP-1 macrophage-derived foam cells according to different transfection groups: oe-NC (transfected with pGMLV-PE2-NC lentiviral vector), oe-CDKN2B-AS1 (transfected with pGMLV-PE2-CDKN2B-AS1 lentiviral vector), sh-NC (transfected with pGMLV-SC6-NC lentiviral vector), sh-CDKN2B-AS1 (transfected with pGMLV-SC6-CDKN2B-AS1 lentiviral vector), and sh-ADAM10 (transfected with pGMLV-SC6-ADAM10 lentiviral vector). THP-1 macrophage-derived foam cells at the logarithmic growth phase were detached and triturated to prepare the cell suspension (5 × 10^4^ cells/mL), seeded into 6-well plates with 2 mL added to each well followed by culture at 37°C overnight. The virus (1 × 10^8^ TU/mL) was added to the cells for infection purposes, and 48 h later, 1 μg/mL puromycin was used to screen the cells after which the infected cells that were stable were collected for subsequent experiments. The cells were subsequently grouped into blank group, methyltransferase SssI (M.SssI) group and DNA methyltransferase inhibitor (5-aza-dc) group. M.SssI and 5-aza-dc reagents were purchased from SHZYSW Biomart Co., Ltd. (Shanghai, China). Specific treatment for the M.SssI group was as follows: Following a 24 h culture and adherence to the wall, the cells were further cultured in M.SssI (final concentration: 2 μmol/L) culture medium, with the medium replaced at regular 24 h intervals. Specific treatment for the 5-aza-dc group was as follows: after being cultured for 24 h, the cells were cultivated in 5-aza-dc (final concentration: 2 μmol/L) culture medium, with the medium replaced every 24 h [44]. Neither M.SssI nor 5-aza-dc was added to the blank group, and the medium was changed at the same time as the M.SssI and 5-aza-dc groups.

### RT-qPCR

The THP-1 cells were collected and lysed by Trizol (Sigma-Aldrich, SF, CA, USA) to extract total cellular RNA. The RNA quality and concentration were determined by an ultraviolet (UV)-visible spectrophotometry. The extracted RNA was subsequently reversely transcribed using a PrimeScript^TM^ RT Reagent Kit (Takara Bio Inc., Otsu, Shiga, Japan). Next, the cDNA was employed as the template, with the target gene then subjected to a fluorescent quantitative PCR operation with reference to the SYBR® Premix Ex Taq^TM^ II (Tli RNaseH Plus) kit (Takara Bio Inc., Otsu, Shiga, Japan). The Real-time fluorescence quantitative PCR was then performed on a Thermal Cycler Dice Real Time System (TP800, Takara Bio Inc., Otsu, Shiga, Japan). The primer sequences for reverse transcription and real-time PCR are depicted in Table 5. The primers utilized for the amplification template were designed by the Primer Express 2.0 software and synthesized by Guangzhou RiboBio Co., Ltd. (Guangzhou, Guangdong, China), and glyceraldehyde-3-phosphate dehydrogenase (GAPDH) was regarded as the internal reference gene. The 2^(-ΔΔCT) indicates the expression of the gene to be tested, Δ CT = CT (target) - CT (ref).

**Table 5:** Primer sequences for RT-qPCR.

Genes	Sequences
CDKN2B-AS1	F: 5'-CTATCCGCCAATCAGGAGGC-3'
	R: 5'-AAAAGGGACACTAGTCCGGC-3'
ADAM10	F: 5'-AAGAAGCTTCCCACAAGGCA-3'
	R: 5'-TGTGTACGCAGAGTATCTAACTGG-3'
GAPDH	F: 5'-ACTAGGCGCTCACTGTTCTC-3'
	R: 5'-ATCCGTTGACTCCGACCTTC-3'

Note: RT-qPCR, reverse transcription quantitative polymerase chain reaction; CDKN, cell-dependent kinase inhibitor; ADAM10, A disintegrin and metalloprotease 10; GAPDH, glyceraldehyde-3-phosphate dehydrogenase; F, forward; R, reverse.

### Cholesterol efflux detection

The THP-1 macrophage-derived foam cells were incubated with 0.2 μCi/mL [^3^H] cholesterol in RPMI 1640 medium containing 10% FBS. Upon reaching approximately 85% confluence, the cells were washed with phosphate buffer saline (PBS) and cultured in lipoprotein-containing serum-free RPMI 1640 medium for 24 h. The cells were then washed again with PBS solution, and subsequently cultured in a fresh medium of 25 μg/mL apoA-I for 6 h. After scintillation lysis of the cells, the [^3^H] cholesterol radioactivity of each sample was measured using a liquid scintillation counter. The cholesterol efflux rate was determined in accordance with the following formula: radiation intensity of culture solution (count per minute, cpm) ÷ total radiation intensity [culture liquid radiation intensity (cpm) + cell lysate radiation intensity (cpm)] × 100%.

### Oil red O staining

THP-1 macrophage-derived foam cells were cultured in 6-well culture plates with sterile coverslips. After treatment, the cells were washed 3 times with PBS, fixed with 50% isopropanol for 1 min, and stained with oil red O staining solution for 10 min. Following hematoxylin staining for 5 min, the color separation as well as blue color development was applied using 1% Hydrochloride. After mounting, the cells were analyzed under a microscope. The intracellular lipids were stained with red and the nuclei were stained with blue. The images were analyzed using an image analysis system and photographed under a microscope.

### HPLC

The THP-1 macrophage-derived foam cells after transfection were collected, washed three times with PBS, and disrupted by sonication in an ice bath. The total protein level was determined in connection with the application of a bicinchoninic acid (BCA) assay. The cell lysate was divided into two portions. One portion was added to an equal volume of freshly prepared 15% potassium hydroxide (KOH) alcohol solution, and stirred at room temperature until the cell lysate was clear for obtaining TC. The other was added to an equal volume of freshly prepared 8.9 mmol/L KOH alcohol in a water bath at 80°C for 1 h in order to obtain FC. The protein was subsequently removed following the addition of 6% trichloroacetic acid. Afterwards, an equal volume of the mixture of n-hexane and isopropanol at the ratio of 4:1 had been added and stirred for 5 min it was subsequently centrifuged at 15°C, 402 ×g for 5 min. The upper organic phase was collected and dried using a vacuum dryer at 65°C. After cooling down, 100 μL of the mixture of isopropanol, n-heptane and acetonitrile at the ratio of 35:13:52 was added in order to dissolve the sample, followed by centrifugation for 5 min at 402 × g to collect the supernatant. Next, 10 μL of the supernatant was collected for HPLC purposes. A non-gradient elution was conducted using a C-18 column with the mixture of isopropanol, n-heptane and acetonitrile as mobile phase at a flow rate of 1 mL/min, and the column temperature was maintained at 4°C. Detection was subsequently performed at 216 nm for 10 min. Cholesterol was quantified by peak area; CE was hydrolyzed by cholesterol esterase, and CE = TC - FC.

### ELISA

The serum levels of IL-1β and TNF-ɑ in THP-1 macrophage culture medium and ApoE-/- mice were determined by ELISA. The IL-1β and TNF-ɑ kits were purchased from Shanghai Bogoo Biological Technology Co., Ltd. (Shanghai, China). The experiments were conducted using the double-antibody sandwich method in strict accordance with the manufacturer’s guidelines. The absorbance (A) value of each well at 450 nm was measured using a microplate reader (Bio-Tek ELx800 Absorbance Microplate Reader). The standard concentration was the abscissa, with the A value regarded as the ordinate. The regression equation of the standard curve was calculated, and the sample A value was substituted into the equation to calculate the sample concentration [45].

### Western blot analysis

Western blot analysis was employed in order to evaluate the ADAM10 protein levels. Total cellular protein was extracted using a high-efficiency radio-immunoprecipitation assay (RIPA) lysis buffer (R0010, Beijing Solarbio Science & Technology Co., Ltd., Beijing, China) in strict accordance with the manual’s instructions. The total protein concentration of each sample was determined using a BCA kit (20201 ES76, Yeasen Company, Shanghai, China). After standardization, the protein was separated by polyacrylamide gel electrophoresis and then blotted onto polyvinylidene fluoride (PVDF) membranes via the wet transfer method. Membrane blockade was conducted using 5% BSA at room temperature for 1 h, followed by incubation with diluted primary rabbit antibody to ADAM10 (ab124695, 1:1000, Abcam Inc., Cambridge, MA, UK) on a shaker at 4°C overnight. The membrane was washed thrice with Tris-buffered saline with tween (TBST) (5 min/time), and incubated with horseradish peroxidase (HRP)-labeled goat anti-rabbit immunoglobulin G (IgG) (ab205718, 1:2000, Abcam Inc., Cambridge, MA, UK) for 1 h at room temperature. The membrane was then washed 6 times with TBST (5 min/time), followed by the addition of the developing solution for image development. The ImageJ 1.48u software (National Institutes of Health, Bethesda, MA, USA) was employed for protein quantification analysis purposes. The protein quantification was performed using the gray value of each protein and the gray ratio of the internal reference GAPDH. Each experiment was repeated three times.

### FISH

FISH was applied in order to detect the localization of CDKN2B-AS1 in THP-1 macrophage-derived foam cells. The cells were treated according to the following as per the instructions of the Ribo^TM^ lncRNA FISH Probe Mix (Red) (Guangzhou RiboBio Co., Ltd., Guangzhou, Guangdong, China). The probe was customized according to CDKN2B-AS1. The specific methodology employed were as follows: The coverslips were placed in a 6-well culture plate, and THP-1 macrophage-derived foam cells were inoculated therein until the cell confluence was approximately 80% after 1 d of culture; the coverslips were removed, washed with PBS and fixed with 1 mL of 4% paraformaldehyde at room temperature; after treatment with 2 μg/mL proteinase K, glycine and acetamidine reagent, the coverslips were added with 250 μL of pre-hybridizations solution and incubated at 42°C for 1 h. After the pre-hybridization solution had been removed, the coverslips were added with 250 μL of the hybridization solution containing the probe (300 ng/mL), and hybridized overnight at 42°C. After three PBST washes, the cells were stained with the 4'-6-diamidino-2-phenylindole (DAPI) staining solution diluted with PBST at a ratio of 1:800 in the 24-well culture plate for 5 min to stain the nucleus. The coverslips were then washed 3 times with PBST (3 min/time). The anti-fluorescent quencher was used for the sealing, and 5 different fields of view were selected under a fluorescence microscope (Olympus Optical Co., Ltd., Tokyo, Japan) for observation, with photomicrographs obtained accordingly [46].

### MS-PCR

MS-PCR was used to detect the methylation status of the ADAM10 promoter. For cell DNA extraction, the genomic DNA extraction kit of Tiangen Biotech Co., Ltd. (Beijing, China) was extracted based on the instructions of the kit. The DNA concentration and purity were determined using a UV spectrophotometer, followed by DNA extraction and storage in a freezer at -80°C for further use. DNA (1 μg) was modified with bisulfite and stored at -80°C for a maximum period of 1 month. The primers for synthesizing methylated and unmethylated ADAM10 genes are illustrated in Table 6, with MSP conducted in accordance with the Herman *et al.* method [47]. The PCR reaction system contained 1 μL of MSP DNA Polymerase (2.5 U/μL), 1.6 μL of deoxynucleoside triphosphate (NTP)s (2.5 mmol/L), 2 μL of 10 × MSP PCR Buffer, 1 μL of forward and reverse primer (10 μmol/L) each, 2 μL of template DNA, and made up to 20 μL with deionized water. Cycle parameters were as follows: 95°C for 5 min, 94°C for 20 s, 60°C for 30 s, 72°C for 20 s, with a total of 35 cycles at 72°C for 5 min. Then 9 μL of MSP product was removed, and 1 μL of 10 × loading buffer was added and electrophoresed on a 2.5% agarose gel containing 0.55 mg/L ethidium bromide. The gel imaging system was used for taking photographs. In the event that the CpG island of the promoter region of the ADAM10 gene was completely methylated, only the methylated primer could amplify the target band; if completely unmethylated, only the unmethylated primer can amplify the target band. In the case of partial methylation, both pairs of primers could amplify the target band. Partial methylation was regarded as a methylation.

**Table 6:** ADAM10 primer sequences.

Primer name	Amplification length	Sequence (5'-3')
Methylated primer	115bp	F: AAAAATTTTTGTTATTTGTGACGT
		R: ATTCCCGTACTACTAAACCTACCG
Unmethylated primer	124bp	F: AAAATTTTTGTTATTTGTGATGT
		R: TTCCCATACTACTAAACCTACCACC

Note: ADAM10, A disintegrin and metalloprotease 10; F, forward; R, reverse.

### ChIP

After treating with 4% formaldehyde (final concentration of formaldehyde was 1%), the collected cells were sonicated, added with the mouse monoclonal antibody to DNMT1 (ab183403, 1:50, Abcam Inc., Cambridge, MA, USA), and bound to the DNMT1-ADAM10 promoter. Protein A Agarose/SaLmon Sperm DNA were then added to the cells, bound to the DNMT1 antibody-DNMT1-ADAM10 promoter complex. The precipitated complex was washed in order to remove some non-specific binding, and eluted to obtain the enriched DNMT1-ADAM10 promoter complex which was decross-linked. The enriched ADAM10 promoter fragment was purified and subjected to PCR analysis, and IgG (ab109489, 1:100, Abcam Inc., Cambridge, MA, USA) was used as a NC [48].

### RIP assay

The binding of CDKN2B-AS1 to DNMT1 protein was detected using a RIP kit (Millipore, Bedford, MA, USA). The cells to be examined and washed with pre-cooled PBS, after which the supernatant was discarded. The cells were lysed with an equal volume of RIPA lysis buffer (P0013B, Beyotime Institute of Biotechnology, Shanghai, China) on an ice bath for 5 min, and centrifuged at 35068 ×g for 10 min at 4°C. The cell extract was co-precipitated by incubation means with the antibody. The specific steps employed were as follows: 50 μL of magnetic beads in each co-precipitation reaction system was washed, resuspended in 100 μL of RIP wash buffer, followed by the addition of 5 μg of antibody for binding based on grouping. The magnetic bead-antibody complex was washed and resuspended in 900 μL of RIP wash buffer and incubated with 100 μL of cell extraction overnight at 4°C. The sample was placed on a magnetic stand to collect the magnetic bead-protein complex. The sample was detached with proteinase K to extract RNA for subsequent PCR detection. The antibody used for RIP was DNMT1 (ab183403, 1:50, Abcam Inc., Cambridge, MA, USA) which was mixed with the complex at room temperature for 30 min, with IgG (ab109489, 1:100, Abcam Inc., Cambridge, MA, USA) as a NC [49].

### RNA pull-down

The RNA fragment was transcribed into a CDKN2B-AS1 RNA fragment *in vitro* using T7 RNA polymerase (Ambion, Company, Austin, TX, USA), treated with the RNeasy Plus Mini Kit (Qiagen company, Hilden, Germany), DNase I (Qiagen company, Hilden, Germany), and purified using the RNeasy Mini Kit. The purified RNA 3' end was biotinylated with a biotin RNA-labeled mixture (Ambion, Company, Austin, TX, USA). Next, 1 μg of labeled RNA was heated to 95°C in RNA structure buffer containing 10 mmol/L Tris (pH = 7), 0.1 mol/L KCl, 10 mmo/L MgCl_2_ for 2 min, incubated on ice for 3 min, then permitted to stand at room temperature for 30 min which allowed the RNA to form a suitable secondary structure. Next, 3 μg of THP-1 macrophage-derived foam cells were added to the cell lysate (Sigma-Aldrich Chemical Company, St Louis, MO, USA) for 1 h at 4°C. The lysate was centrifuged at 12000 ×g at 4°C for 10 min, with the supernatant collected and transferred to an RNase-free centrifuge tube. Next, 400 ng of biotinylated RNA was added to a 500 μL of RIP buffer and mixed with cell lysate for 1 h at room temperature. Streptavidin magnetic beads were added to each binding reaction and incubated for 1 h at room temperature. Finally, the cells were washed 5 times with RIP buffer, and added with 5 × loading buffer. After incubation at 95°C for 5 min, the eluted DNMT1 protein was detected by Western blot analysis. The antibody used was mouse monoclonal antibody to DNMT1 (ab183403, 1:1000, Abcam Inc., Cambridge, MA, USA).

### Establishment and identification of ApoE-/- atherosclerosis mouse model

A total of 60 healthy male ApoE-/- mice of specific pathogen-free (SPF) grade (aged 8 - 10 weeks and weighing 20 - 30 g) purchased from Nanjing Better Biotechnology Co., Ltd. (Nanjing, Jiangsu, China) were recruited in order to establish atherosclerosis mouse models. The success rate of modeling was 80% with 48 mice confirmed to be successfully established. Twelve healthy male C57BL/6J mice of SPF grade (aged 8 - 10 weeks and weighing 20 - 30 g) were purchased from Nanjing Better Biotechnology Co., Ltd. (Nanjing, Jiangsu, China) as regarded as the normal control. The experimental mice were housed in an SPF animal laboratory with a humidity of 50% to 60% and a temperature of 22 to 25 °C. After 7 d of adaptive feeding, the mice in the model group were placed on a high-fat diet (21% fat and 0.15% cholesterol) for 10 weeks, while the C57BL/6J mice in the control group remained on a normal diet. Successful modeling was based on the following aspects: (1) Physiological state: mice with dark fur, pale and dull claws exhibiting fatigue, sluggish appetite, and slow actions; (2) Pathological examination: a large number of atheromatous plaques detected in the arteries, formation and accumulation of a large number of foam cells with a thickened aortic intima, disordered tissue arrangement, as well as distinct plaque instability; (3) Abnormal levels of TC, TG, HDL, and LDL in blood lipid were detected [37]. The mice in the control group were noted to be lively and active with smooth fur and normal appetite and mental condition, and their tissues were neatly arranged without plaque. After successful atherosclerosis mouse modelling of ApoE^-/-^, the mice were then placed on a normal diet for 4 weeks. The ApoE^-/-^ mice were then divided into 4 groups (12 in each group): M-oe-NC, M-oe-CDKN2B-AS1, M-oe-CDKN2B-AS1 + oe-ADAM10 and M-oe-ADAM10 groups. The RAW 264.7 macrophage-derived foam cells purchased from the Basic Medical Cell Center of the Institute of Basic Medical Sciences of the Chinese Academy of Medical Sciences (Beijing, China) (http://www.crcpumc.com/pr.jsp?keyword=THP1&_pp=0_312) in the logarithmic growth phase were detached with trypsin and triturated to prepare a cell suspension of 5 × 10^4^ cells/mL. The suspension was seeded into a 6-well plate with 2 mL per well, and cultured at 37°C overnight. The virus (1 × 10^8^ TU/mL) was then added to the cells for infection purposes, allowing for collection of more stable infected cell lines. The virus-infected cell suspension was then injected into each group of mice. After the first week, the mice were injected twice during the first week (injection dose: 80 mL/kg cell suspension dissolved in 0.2 mL of saline), and once a week for the next 3 weeks, for a total of four weeks.

### Mouse specimen processing

On the day prior to blood collection, the mice in each group were fasted for 12 h. After anesthesia with 3% pentobarbital sodium, the blood was collected from the ocular venous plexus of the mice. After settling, the serum samples were obtained by centrifugation at 1610 ×g for 15 min at 4°C. The mice were sacrificed after blood collection, and the ventral midline incision was performed to separate the sternum and diaphragm from the blunt. The heart and kidneys were isolated, with the thoracic and abdominal aorta exposed. The extravascular membrane was dissected, after which the tissues were isolated and the entire aorta was removed. After multiple PBS washes, the entire aorta was fixed with 4% paraformaldehyde for 24 h for subsequent experiments.

### Blood lipid level test

The serum levels of TC, TG, HDL and LDL were evaluated using an automatic biochemical analyzer (AD-VIA-2400, Siemens Ltd., Erlangen, Germany) with biochemical detection reagent (01218LH, Beijing Leadman, Beijing, China).

### Oil red O staining of aortic

The mice were fasted 1 day prior to execution by a subcutaneous injection with 3% sodium pentobarbital at a dose of 50 mg/kg. The aortic arch to the branch of the abdominal aorta was with the aorta then longitudinally dissected. The aorta was squashed, stained with oil red O staining solution for 15 min, soaked for 20 min with 70% alcohol, and rinsed with distilled water for 30 min. The arteries were then placed on a white plate, photographed and analyzed quantitatively using the Image-Pro Plus 6.0 software (Media Cybernetics, Bethesda, MD, USA) for plaque area. The tissues stained with red were considered to be the atherosclerotic lesions.

### HE staining

The formaldehyde-fixed specimens were rinsed with distilled water for 1 h, and then fully soaked in the following reagents, 70% alcohol for 24 h, 80% alcohol for 24 h, 95% alcohol (I) for 30 min, 95% alcohol (II) for 30 min, 100% alcohol (I) for 30 min, 100% alcohol (II) for 30 min, the mixture of 100% alcohol and xylene mixture at the ratio of 1:1 for 20 min, xylene (I) for 20 min, xylene (II) for 20 min, soft wax for 10 min, and hard wax for 10 min. The samples were then embedded in paraffin blocks and lightly sliced into a thickness of 5 μm with a controlled speed. The slices were then gently spread on a glass slide, baked at 60°C for 1 h, and stored at room temperature. The paraffin-embedded sections were then routinely dewaxed with xylene, stained with hematoxylin for 5 - 10 min, rinsed under tap water, and differentiated using 1% aqueous hydrochloric acid. The sections were subsequently stained with eosin solution for 1 - 2 min and rinsed thoroughly under tap water. Finally, the sections were dehydrated with ethanol, cleaned, and sealed with neutral resin. A fluoroscope (Olympus, Tokyo, Japan) was employed in order to observe the morphological structure of the blood vessels and plaques, with ImageJ image processing software was used for plaque area measurement and image collection purposes.

### *In vivo* cholesterol efflux detection

Initially, ox-LDL with a final concentration of 50 μg/mL was added into a 15 mL centrifuge tube and water-bathed with [^3^H] cholesterol at a concentration of 5 μCi/mL at 37°C for 30 min. After the above mixture had been added into the DMEM medium, the RAW 264.7 macrophage-derived foam cells were transfected with oe-NC, oe-CDKN2B-AS1 alone, oe-CDKN2B-AS1 and oe-ADAM10, or oe-ADAM10 alone and incubated at 37°C for 48 h. After centrifugation and resuspension, the cell number was adjusted to 10 × 10^9^ cells/L, with the radioactivity of the injection suspension set at 6.2 × 10^6^ cpm/mL. Next, 0.5 mL of isotope-labeled cell suspension was injected into each mouse through the abdominal cavity. After 48 h, the serum of the mice was collected. The blood samples were placed on ice for 1 h and centrifuged at 258 ×g for 5 min. A total of 20 μL of serum was taken to calculate the blood radioactivity using a flash counter (FJ22107P, State-owned 262 nuclear instrument factory, Xi'an, Shaanxi, China).

### Statistical analysis

All data were analyzed by SPSS 21.0 statistical software (IBM Corp. Armonk, N.Y., USA). Measurement data were expressed in the form of mean ± standard deviation. All data were subjected to normal distribution and homogeneity of variance tests. In the event that data conformed to normal distribution and homogeneity of variance, comparisons between two groups were analyzed by unpaired *t*-test while comparisons among multiple groups were performed by one-way analysis of variance (ANOVA). If the data did not conform to normal distribution or homogeneity of variance, the data were analyzed using rank-sum test. A value of *p* < 0.05 was considered to be indicative of statistical significance.
